# The ancillary protein 1 of *Streptococcus pyogenes* FCT-1 pili mediates cell adhesion and biofilm formation through heterophilic as well as homophilic interactions

**DOI:** 10.1111/j.1365-2958.2012.07987.x

**Published:** 2012-02-10

**Authors:** Marco Becherelli, Andrea G O Manetti, Scilla Buccato, Elisa Viciani, Laura Ciucchi, Giulia Mollica, Guido Grandi, Imma Margarit

**Affiliations:** Novartis Vaccines and DiagnosticsVia Fiorentina 1, 53100 Siena, Italy

## Abstract

Gram-positive pili are known to play a role in bacterial adhesion to epithelial cells and in the formation of biofilm microbial communities. In the present study we undertook the functional characterization of the pilus ancillary protein 1 (AP1_M6) from *Streptococcus pyogenes* isolates expressing the FCT-1 pilus variant, known to be strong biofilm formers. Cell binding and biofilm formation assays using *S. pyogenes* in-frame deletion mutants, *Lactococcus* expressing heterologous FCT-1 pili and purified recombinant AP1_M6, indicated that this pilin is a strong cell adhesin that is also involved in bacterial biofilm formation. Moreover, we show that AP1_M6 establishes homophilic interactions that mediate inter-bacterial contact, possibly promoting bacterial colonization of target epithelial cells in the form of three-dimensional microcolonies. Finally, AP1_M6 knockout mutants were less virulent in mice, indicating that this protein is also implicated in GAS systemic infection.

## Introduction

The discovery of covalently linked pilus polymers on the surface of an increasing number of Gram-positive pathogens has encouraged many research efforts aimed at elucidating their structure, mechanism of assembly and biological role (Ton-That and Schneewind, 2004; Telford *et al*., 2006; Kreikemeyer *et al*., 2011). Similar pictures have emerged, despite the high inter- and intra-species sequence variability of their protein components. First, Gram-positive pili share a common mechanism of assembly, according to which individual subunits are linked by dedicated sortases catalysing the formation of inter- and intra-molecular isopeptide bonds, and the assembled polymer is attached to the peptidoglycan via a cell wall-anchoring sortase (Ton-That and Schneewind, 2003; Kang *et al*., 2007). The pilus shaft is constituted by the Backbone Protein (BP), while one or two Ancillary Proteins (AP1, AP2) may be present at the pilus tip, its base or along the pilus (Budzik *et al*., 2008; Nobbs *et al*., 2008; Mandlik *et al*., 2008a; Hilleringmann *et al*., 2009; Smith *et al*., 2010). Additional common themes have emerged from functional studies, indicating a role of pili in host–pathogen interactions by mediating bacterial adhesion to epithelial cells and biofilm formation (Mandlik *et al*., 2008b; Kreikemeyer *et al*., 2011). Furthermore, several studies using animal models have confirmed a role in bacterial pathogenesis (Barocchi *et al*., 2006; Nallapareddy *et al*., 2006; Maisey *et al*., 2008).

A relevant human pathogen bearing pilus appendages is *Streptococcus pyogenes* (Group A *Streptococcus*, GAS). GAS colonizes the human pharynx and the skin, causing self-limiting infections like pharyngitis or impetigo. It can also trigger life-threatening diseases, like necrotizing fasciitis and toxic shock, and important autoimmune sequelae like rheumatic heart disease and glomerulonephritis.

GAS pili are encoded in the FCT (Fibronectin, Collagen, T antigen) genomic region, of which nine variants with different gene organization and sequences have been identified so far, each present in a subset of M serotypes (Kratovac *et al*., 2007; Falugi *et al*., 2008). The pilus structures of GAS FCT-2 isolates were shown to contribute to biofilm formation and bacterial adhesion to epithelial cells (Abbot *et al*., 2007; Manetti *et al*., 2007; Crotty Alexander *et al*., 2010). Binding of different GAS pilus types to the scavenger receptor gp340 was also shown to mediate bacterial aggregation in saliva (Edwards *et al*., 2008). More recent studies identified the AP1 ancillary protein as the main FCT-2 pilus adhesin (Smith *et al*., 2010). Finally, inactivation of AP1 in a FCT-3 M53 background determined a loss of virulence in a mouse model of superficial skin infection (Lizano *et al*., 2007).

The high divergence between different GAS pilus types poses the question whether this variability affects their biological function. In a previous study, we addressed this issue by investigating the capacity of isolates belonging to different FCT types to form biofilms under diverse environmental conditions. We observed that a subset of GAS FCT types formed biofilms only under acidic conditions and that this capacity was directly related to an enhanced pilus expression at low pH (Koller *et al*., 2010; Manetti *et al*., 2010). Remarkably, FCT-1 isolates (M6 and M119 serotypes) formed the strongest biofilms among the different GAS FCT types, owing to their capacity to highly express pili irrespective of any pH condition.

In the present study, we investigated the specific role of the AP1 component of FCT-1 M6 pili in bacterial cell adhesion, biofilm formation and survival during infection. We observed that this pilus component displayed high affinity to epithelial cells and that an isogenic knockout mutant deprived of the *ap1* gene was impaired in its capacity to adhere to epithelial cells. We provide experimental evidence for the establishment of homophilic interactions between AP1 pilus proteins from different FCT-1 bacteria, mediating the formation of large three-dimensional microcolony aggregates. Finally, the *ap1* knockout mutant showed a decreased capacity to survive in blood and was less virulent than the wild-type strain in a mouse model of GAS infection.

## Results

### The FCT-1 M6 pilus ancillary protein mediates epithelial cell adherence

**Fig 1:**
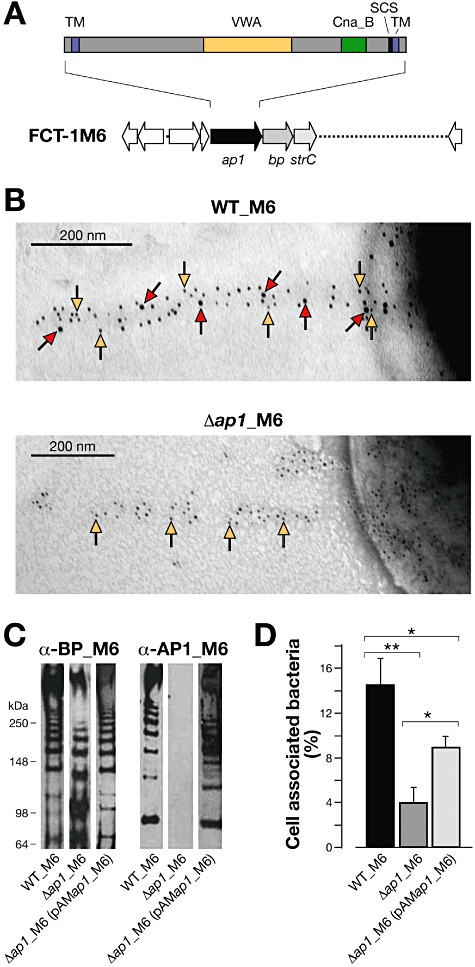
Structural organization and adhesive properties of the ancillary protein AP1_M6 of FCT-1 pili. A. Schematic representation of the FCT-1 pilus island genetic organization and of the structural domains of AP1_M6, including the hydrophobic transmembranes (TM), the Von-Willebrand factor type A domain (VWA), the Cna_B domain and the sortase cleavage site (SCS). B. Immunoelectron microscopy image taken at 30× magnification after double immunogold labelling of FCT-1 M6 pili from wild type (WT_M6) and its isogenic Δ*ap1*_M6 derivative with rabbit polyclonal anti-rBP_M6 and mouse anti-rAP1_M6 antibodies, followed by gold-labelled anti-rabbit IgG (particle size 5 nm, yellow arrows) and anti-mouse IgG antibodies (10 nm, red arrows). C. Immunoblot of cell wall fractions of WT_M6, Δ*ap1*_M6 and the complemented strain Δ*ap1*_M6(pAM*ap1*_M6). Total protein amounts of the different extracts loaded on the gels were normalized using antibodies to protein Spy0269. Transferred extracts were incubated with specific mouse sera raised against rBP_M6 and rAP1_M6 proteins. D. Binding of WT_M6, Δ*ap1*_M6 and Δ*ap1*_M6(pAM*ap1*_M6) to A549 cells (moi 100:1) after 30 min of incubation. Histograms represent the percentage of cell-associated versus total bacteria. Results are presented as the mean and standard deviation values of three independent experiments run in triplicate wells. Significant differences between strains calculated by Student's *t*-test are shown by asterisks **P < 0.01, *P < 0.05.

Unlike other GAS pilus types, FCT-1 pili contain exclusively two components, the pilus Backbone Protein Tee 6 (herein BP_M6) and the ancillary protein FctX (herein AP1_M6) (Bessen and Kalia, 2002) (Fig. 1A). The two proteins display less than 25% identity to BP and AP1 variants from other FCT types (Kratovac *et al*., 2007; Falugi *et al*., 2008). *In silico* analysis of AP1_M6 revealed a Cna_B domain shared by several adhesins forming stabilizing isopeptide bonds (Kang *et al*., 2007) as well as a possible Von-Willebrand factor type A domain (VWA) spanning amino acids 439–638, also identified in pilus components from *S. pneumoniae* (Nelson *et al*., 2007), *Corynebacterium diphtheriae* (Mandlik *et al*., 2007) and *Streptococcus agalactiae* (GBS) (Konto-Ghiorghi *et al*., 2009) (Fig. 1A). In the latter case, the VWA domain was directly implicated in pilus-mediated bacterial adhesion to epithelial cells.

To investigate the contribution of AP1_M6 to epithelial cell adhesion, we produced an isogenic deletion mutant of the *ap1* gene in the background of a GAS FCT-1 M6 isolate. Electron micrographs of pili expressed by wild type (WT_M6) and the Δ*ap1* mutant (Δ*ap1*_M6) are presented in Fig. 1B. Wild-type pili were decorated with 5 and 10 nm gold nanoparticles corresponding to the backbone and the AP1 protein, while pili exclusively composed of the backbone protein were observed for Δ*ap1*_M6. Western blot analysis of wild-type bacterial surface extracts using anti-AP1_M6 or anti-BP_M6 specific sera revealed a ladder of bands corresponding to pilus polymers (Fig. 1C). Analysis of Δ*ap*1_M6 extracts confirmed the absence of AP1_M6 bands and revealed the presence of BP polymers with slightly different migration and lower amount than the wild type. Finally, the complemented strain Δ*ap1*_M6 (pAM*ap1*_M6) obtained by transforming Δ*ap1*_M6 with a plasmid containing the *ap1*_M6 gene under a constitutive *S. agalactiae* promoter (Buccato *et al*., 2006), expressed pilus polymers constituted by AP1 and BP to a similar extent as WT_M6 when grown in the presence of the antibiotic used as plasmid selective marker. These data suggested that AP1 is not essential for the formation of BP polymers, but in part contributes to the polymerization process. Flow cytometry (FACS) analysis of wild-type and the knockout mutant bacteria with antibodies raised against recombinant AP1_M6 (rAP1_M6) and BP_M6 (rBP_M6), essentially confirmed the complete absence of AP1 and the lower surface exposure of BP in the mutant strain, while antibodies raised against Emm6 protein and C5a peptidase (ScpA) indicated similar expression levels of these two relevant GAS virulence factors (Fig. S1).

In control experiments, Δ*ap1*_M6 and the complemented strain compared with the wild-type parental strain, displayed identical growth rates in different media (data not shown). We subsequently analysed the adhesive phenotypes of wild type, Δ*ap1*_M6, and the complemented mutant by incubating the bacteria for 30 min with A549 pulmonary epithelial cell monolayers, followed by extensive washing, cell lysis and bacterial enumeration. As shown in Fig. 1D, we observed a fivefold difference between the number of wild type and Δ*ap1*_M6 associated to cells. Adhesion could be partially but not completely restored in the complemented mutant strain Δ*ap1*_M6 (pAM*ap1*_M6), probably due to the plasmid instability problems that were encountered in the absence of antibiotic selective pressure.

**Fig 2:**
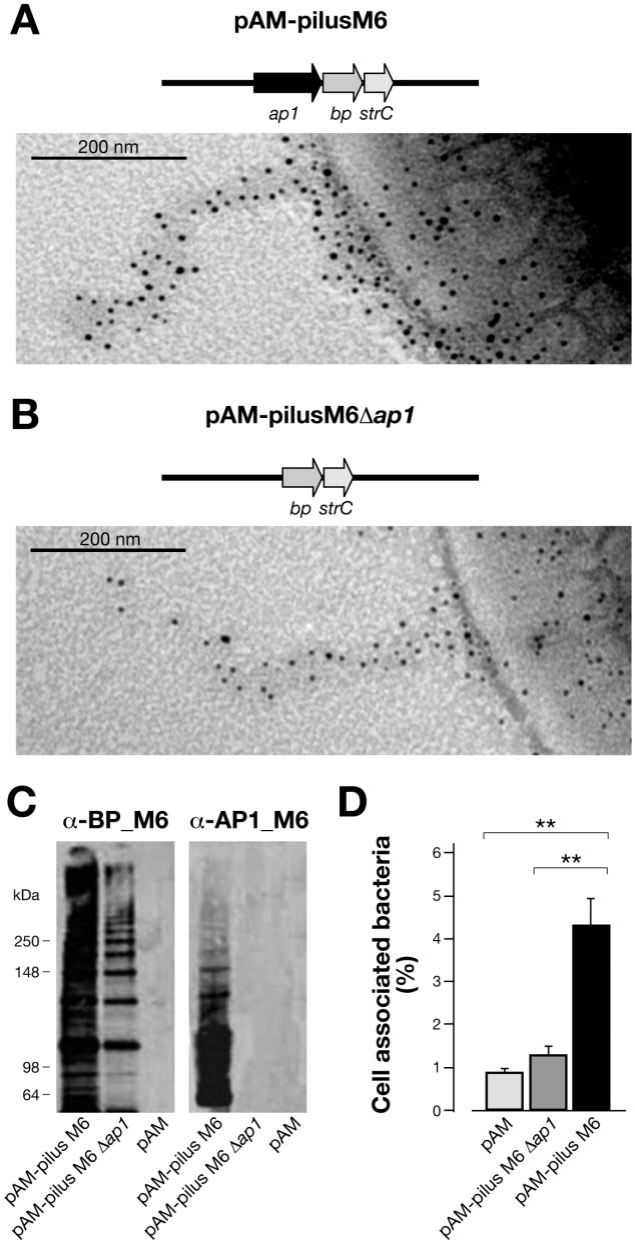
Adhesive properties of *L. lactis* expressing the FCT-1 M6 pilus operon and its Δ*ap1*_M6 derivative. A. Immunoelectron microscopy image of a *L. lactis* recombinant strain harbouring the entire FCT-1 M6 pilus operon. B. Immunoelectron microscopy image of a *L. lactis* recombinant strain harbouring the FCT-1 M6 pilus operon deprived of the *ap1* gene. In both cases, bacteria were first incubated with polyclonal sera raised against rBP_M6, and then labelled with secondary antibodies conjugated with 5 nm gold particles. Pictures were taken at 60× magnification. C. Immunoblot analysis of *L. lactis* cell wall extracts incubated with antisera raised against rBP_M6 and rAP1_M6 pilus proteins. D. Binding of *L. lactis* recombinant strains to A549 lung epithelial cells (moi 20:1) after 30 min of incubation, expressed as percentage of cell-associated versus total bacteria. Results are presented as the mean and standard deviation values of three independent experiments run in triplicate wells. Significant differences between strains (**P < 0.01) were estimated using the Student's *t*-test.

The role of AP1_M6 in cell adhesion was further investigated using recombinant *Lactoccocus lactis* expressing either the entire FCT-1 pilus operon carrying *ap1*, *bp* and *srtC* genes (pAM-pilusM6), or the same locus deprived of the *ap1* gene (pAM-pilusM6Δ*ap1*) under the above mentioned GBS constitutive promoter. The genes inserted in the two constructs are shown in Fig. 2A and B. The two recombinant clones produced pilus structures, as observed by electron micrographs of whole bacteria labelled with anti-BP antibodies (Fig. 2A and B). Western blot analysis of surface extracts showed that the amount of high-molecular-weight pilus polymers was more enhanced in *Lactococcus* expressing the entire locus (pAM-pilusM6) than in the clone lacking the *ap1* gene (pAM-pilusM6Δ*ap1*, Fig. 2C), confirming that AP1_M6 in part contributes to pilus polymerization. The two recombinant clones were compared with *L. lactis* carrying the empty vector for their capacity to bind A549 cells. As shown in Fig. 2D, after 30 min of incubation the number of cell-associated bacteria expressing BP_M6 pili (pAM-pilusM6Δ*ap1*) was similar to that of bacteria carrying the empty vector (pAM), indicating that the backbone pilus protein was not directly involved in cell adhesion. On the other hand, this number significantly increased by about fourfold in the case of *Lactococcus* expressing pili that also contained AP1_M6 (pAM-pilusM6), suggesting that this pilus ancillary protein could indeed play a role in pilus-mediated cell adhesion.

**Fig 3:**
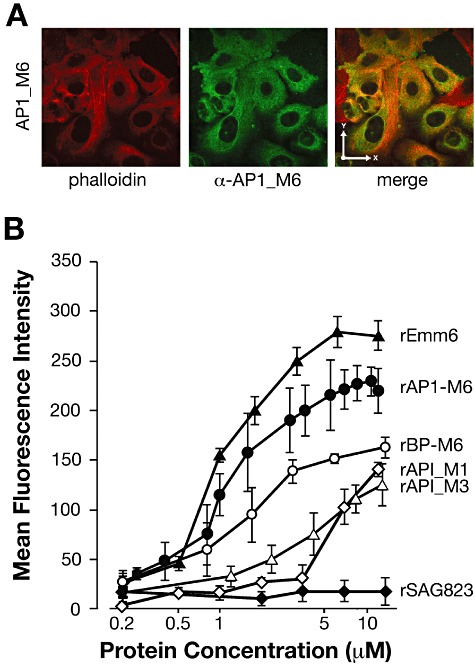
Binding of rAP1_M6 and other recombinant proteins to epithelial cells. A. Confocal microscopy analysis of rAP1_M6 protein stained with specific antibodies (green) binding to epithelial cells (labelled with phalloidin, red); the *x*- and *y*-axis arrows in the merged image indicate 27 µm dimensions. B. FACS analysis of GAS proteins binding to A549 cells. Cells were incubated with increasing concentrations of the following recombinant proteins: pilus ancillary proteins rAP1_M6, rAP1_M1 and rAP1_M3, the pilus backbone rBP_M6, the rEmm6 protein and, as negative control, rSAG0823 from *S. agalactiae*. Cell-bound proteins were detected with specific antibodies and fluorescent secondary antisera, followed by flow cytometry analysis. Results are presented as the mean and standard deviation values of three independent experiments.

**Fig 6:**
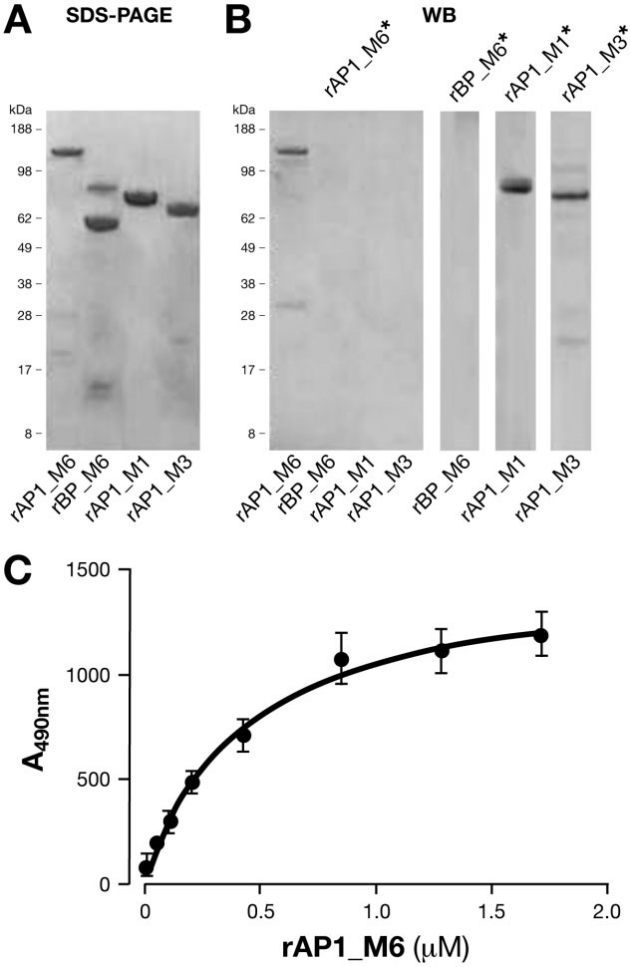
Biochemical analysis of protein–protein interactions involving pilus proteins. A. SDS-PAGE analysis of recombinant pilus proteins rAP1_M6, rBP_M6, rAP1_M1 and rAP1_M3; lane 2 contains a main band of 62 kDa corresponding to rBP_M6 and a secondary band of about 90 kDa, which corresponds to an impurity from *E. coli*. B. Far Western blot analysis using biotinylated rAP1_M6 (rAP1_M6*) as probe against the recombinant proteins shown in (A); Far Western blot assay of biotinylated rBP_M6*, rAP1_M1* and rAP1_M3* as probes against the respective pilus proteins loaded on SDS-PAGE. C. ELISA saturation kinetic analysis of biotinylated rAP1_M6* protein bound to immobilized unlabelled rAP1_M6. All experiments were conducted in triplicate with reproducible results, and mean and standard deviation values are reported.

The capacity of a recombinant form of the full-length AP1_M6 protein (rAP1_M6, amino acids 1–1068) to bind A549 epithelial cells was visualized by confocal microscopy analysis, where the recombinant protein stained by specific antibodies colocalized with cells stained by phalloidin (Fig. 3A). To further confirm AP1_M6 as a pilus adhesin, the cell binding property of rAP1_M6 was compared with that of the pilus backbone protein (rBP_M6), the FCT-3 M3 variant of AP1 (rAP1_M3), the FCT-2 M1 AP1 (rAP1_M1), the Emm6 protein (rEmm6) and a non-adhesive protein from Group B *Streptococcus* (rSAG0823). An SDS-PAGE analysis of the purified pilus proteins is shown in Fig. 6A. Increasing concentrations of each purified protein were incubated with cell monolayers and binding was revealed by FACS analysis using specific polyclonal antibodies. As shown in Fig. 3B, all pilus proteins bound to A549 cells in a dose-dependent manner. In particular, rAP1_M6 displayed a binding curve similar to that obtained with rEmm6 and more steep than rBP_M6, rAP1_M1 and rAP1_M3. A Scatchard analysis of the curve obtained from three independent cell binding experiments with rAP1_M6 was used to derive an apparent cell binding affinity (*K*_D_) of about 8.7 ± 3.2 × 10^−7^ M, as the concentration determining the saturation of 50% of the receptors present on the cells.

Finally, we investigated whether the VWA domain present in AP1_M6 was directly involved in the adhesive property of the protein. Three fragments of AP1_M6 were expressed and purified from *E. coli*, i.e. an N-terminal portion spanning residues 1 to 499, a C-terminal fragment from amino acids 505 to 1068 including the VWA and the Cna_B domains, and a shorter C-terminal fragment from amino acids 964 to 1068 containing exclusively the Cna_B domain (Fig. S2A). The cell binding capacity of increasing concentrations of each of the three fragments was compared by FACS analysis to that of full-length rAP1_M6, using antibodies raised against each of the polypeptides. As shown in Fig. S2B, the large C-terminal fragment containing both VWA and Cna_B domains displayed similar cell binding affinity to the full-length protein, while the N-terminal fragment and the smaller C-terminal portion containing exclusively the Cna_B domain showed a lower binding capacity. The data suggest that the VWA domain of AP1_M6 contributes to the adhesive properties of the FCT-1 pilus accessory protein.

### AP1_M6 mediates biofilm and microcolony formation

**Fig 4:**
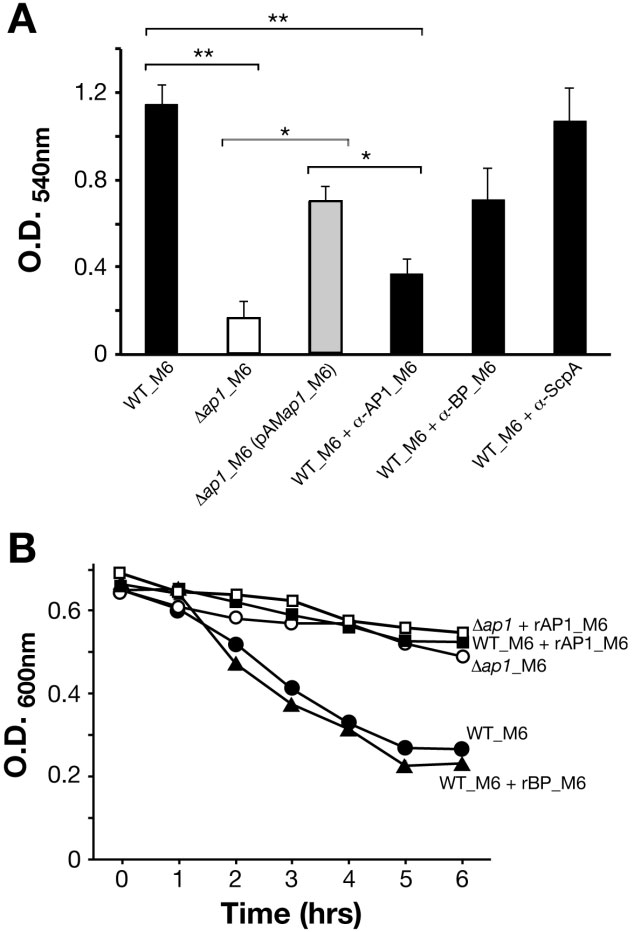
Role of AP1_M6 in biofilm formation and bacterial aggregation. A. Crystal violet biofilm formation assay. GAS WT_M6, Δ*ap1*_M6 and complemented bacteria were grown for 10 h in 24-well plates, washed with PBS and stained with crystal violet. Experiments with WT_M6 were also conducted in the presence of purified mouse polyclonal antibodies raised against rAP1_M6 and rBP_M6 pilus components, and against rScpA as control. The mean and SD values of three independent experiments run in triplicate wells are reported. Significant differences between strains calculated by Student's *t*-test are shown by asterisks, **P < 0.01, *P < 0.05. B. Sedimentation of GAS WT_M6 grown in the presence or absence of 1 µM rAP1_M6 or rBP_M6, compared with that of Δ*ap1*_M6 mutant strain. Bacteria were grown for 16 h, bacterial precipitates were suspended by mixing, and OD values were at 600 nm measured at different time intervals under static conditions; the presented data derive from one of three experiments with reproducible results.

We had previously demonstrated that FCT-1 isolates are very strong biofilm formers, and that this capacity is directly related to high pilus expression (Manetti *et al*., 2010). To investigate the specific contribution of AP1_M6 to bacterial biofilm formation, wild-type M6 and its Δ*ap1*_M6 mutant derivative were incubated for 10 h on polystyrene plates, and biofilm production was assessed by crystal violet staining. As shown in Fig. 4A, wild-type bacteria produced strong biofilms, whereas Δ*ap1*_M6 was severely impaired in its biofilm forming capacity. The complemented mutant, which as already pointed out had a tendency to lose the plasmid carrying the complementing gene in the absence of antibiotics, partially recovered the capacity to build biofilm structures.

Moreover, incubation of wild type with polyclonal antibodies raised against rAP1_M6 abolished biofilm formation, whereas antibodies to rBP_M6 had a lower effect, and no inhibition was observed with anti-ScpA as control (Fig. 4A).

To investigate whether AP1_M6 could play an active role in the establishment of inter-bacterial interactions leading to biofilm maturation, wild type and Δ*ap1*_M6 were grown overnight, bacterial precipitates were suspended by mixing, and OD values were measured at different time intervals under static conditions. As shown in Fig. 4B, wild-type bacterial aggregates immediately started to sediment and the OD_600_ dropped from 0.6 to 0.3 after 5 h. Conversely, the Δ*ap1*_M6 mutant maintained a constant OD value. Remarkably, addition of rAP1_M6 protein prevented wild-type sedimentation, while the same inhibitory effect was not observed using rBP_M6. The above results led us to hypothesize that protein–protein interactions taking place between distinct AP1_M6 molecules could promote inter-bacterial contact, aggregation and biofilm formation on abiotic and biological surfaces.

### AP1_M6 establishes homophilic interactions

**Fig 5:**
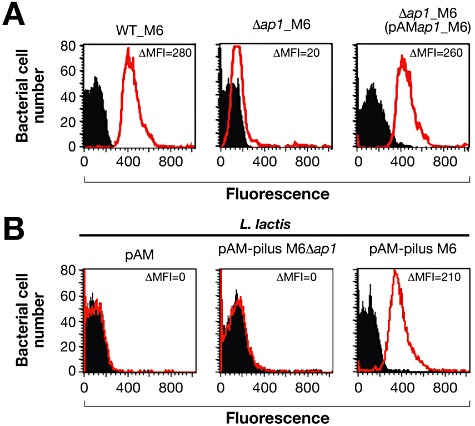
Flow-cytometry analysis of the interaction between recombinant rAP1_M6 and GAS or *Lactococcus* bacteria. A. FACS analysis of GAS WT_M6, its deletion mutant Δ*ap1*_M6 and the complemented strain Δ*ap1*_M6(pAM *ap1*_M6). B. FACS analysis of *L. lactis* carrying either an empty vector (pAM), or a plasmid containing the FCT-1 M6 pilus operon (pAM-pilus M6) or the same operon except for a deletion in the *ap1_M6* gene. In all cases bacteria were incubated with 5 µg of biotinylated rAP1_M6 and binding of the protein to the cells was revealed by R-Phycoerythrin-conjugated Streptavidin. Black histograms indicate staining of bacteria with streptavidin alone, while red histograms correspond to bacteria incubated with rAP1_M6 protein and then streptavidin. Binding was calculated by subtracting the mean fluorescence of bacteria stained with biotinylated rAP1_M6 protein from that of the unstained bacteria (ΔMFI). The average ΔMFI value from three independent experiments is shown.

The ability of AP1_M6 to establish homophilic interactions was investigated by incubating wild-type bacteria, the Δ*ap1*_M6 mutant and its complemented derivative Δ*ap1*_M6 (pAM*ap1*_M6) with 5 µg of biotinylated rAP1_M6, followed by fluorescently labelled streptavidin and flow cytometry analysis. As shown in Fig. 5A, labelled rAP1_M6 bound to wild type (WT) and to the complemented Δ*ap1*_M6 (pAM*ap1*_M6) bacteria, while binding to Δ*ap1*_M6 was completely abolished. The data suggested that soluble rAP1_M6 interacted exclusively with its homologous counterpart present on the bacterial surface, and not with other GAS surface proteins. To confirm the specificity of the homophilic interaction, we incubated biotinylated rAP1_M6 with the three obtained *Lactococcus* derivatives, carrying either an empty vector, or the entire pilus operon, or the operon deprived of the *ap1* gene. Also in this case, the recombinant protein bound exclusively to bacteria expressing AP1_M6 on their surface (Fig. 5B).

The homophilic interactions between AP1_M6 molecules were further assessed by Far Western blot, using biotinylated rAP1_M6 (rAP1_M6*) as a probe against the same unlabelled protein loaded onto SDS-PAGE (Fig. 6A) and then immobilized on nitrocellulose (Fig. 6B). Possible heterologous interactions between rAP1_M6* and immobilized rBP_M6 or rAP1 ancillary proteins from other FCT types (rAP1_M1 and rAP1_M3) were also investigated. As shown in Fig. 6B, biotinylated rAP1_M6* bound specifically to its immobilized counterpart, while no binding was detected with rBP_M6, rAP1_M1 or rAP1_M6, all sharing less than 25% identity with AP1_M6. We subsequently asked whether the capacity to establish homophilic interactions was a feature specific to AP1_M6 or was shared by other pilus proteins. To address this issue, we performed similar Far Western blot experiments using biotinylated rBP_M6*, rAP1_M1* and rAP1_M3* to probe the corresponding unlabelled proteins (Fig. 6B). Remarkably, the two rAP1 variants gave similar results to rAP1_M6, while no interactions involving rBP_M6 were detected.

Figure 6C shows a dose-dependent saturable binding between increasing concentrations of biotinylated rAP1_M6* and a fixed amount of unlabelled protein immobilized on ELISA plates. The data were fitted using the equation *A* = Amax [*L*] *K*_A_/(1 + *K*_A_ [*L*]), where *A* corresponds to the absorbance at 490 nm, [*L*] is the molar concentration of biotinylated ligand, and *K*_A_ corresponds to the affinity association constant. An apparent dissociation constant (*K*_D_) of 4.46 ± 0.65 × 10^−7^ M was calculated as the reciprocal value of the *K*_A_.

The obtained results suggest a general mechanism whereby GAS pili mediate aggregation between bacteria of the same pilus type and biofilm formation by means of homophilic interactions between their AP1 components.

### AP1_M6 contributes to bacterial virulence in a systemic mouse model of infection

**Fig 7:**
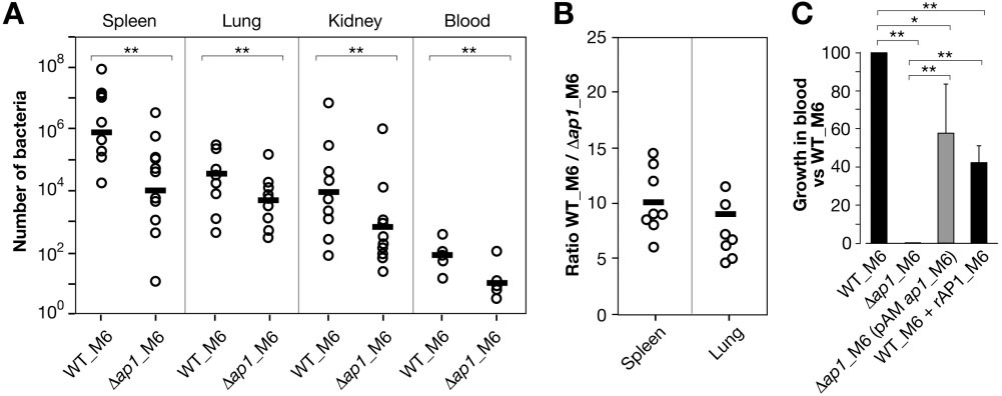
Analysis of bacterial infection in mice and blood survival of WT_M6 and Δ*ap1*_M6 strains. A. Number of bacteria obtained after plating dissected and homogenized spleens, lungs, kidneys (total number of cfu) and blood (cfu ml^−1^) collected 24 h post intraperitoneal infection of mice using 2 × 10^7^ cfu of GAS WT_M6 or Δ*ap1*_M6 strains (12 animals per strain, in three independent experiments with four animals each). Each circle represents the number of bacteria recovered in the organ of one mouse, and the average number of bacteria from all mice is indicated by a bar. Differences were statistically significant in all organs (**P < 0.01, *U*-test). B. Competitive index of GAS WT_M6 versus Δ*ap1*_M6 strains. Eight mice (in two independent experiments with four animals each) were infected intraperitoneally with 10^7^ cfu of WT_M6 and 10^7^ cfu of Δ*ap1*_M6. After 24 h, bacteria were recovered from the lungs and spleens of sacrificed animals and plated. Wild-type bacteria were discriminated from mutant strains by colony blot analysis using antibodies to rAP1_M6 protein. Each circle represents the ratio between the number of cfu of the two strains in the organ of one mouse, and the average ratio is indicated by a bar. C. Survival of GAS WT_M6 with or without rAP1_M6, of the deletion mutant Δ*ap1*_M6, and of the complemented strain Δ*ap1*_M6(pAM*ap1*_M6) in whole blood from rabbits. For each strain, the average relative growth rate (cfu at time 5 h divided by cfu at time 0) versus that of wild type (to which we assigned a value of 100) obtained from three different experiments, and its standard deviation, are reported. Significant differences between strains were calculated by Student's *t*-test, **P < 0.01, *P < 0.05.

We subsequently evaluated the role of AP1_M6 in promoting GAS systemic virulence by challenging intraperitoneally 12 CD1 mice with wild-type or Δ*ap1*_M6 strains. After 24 h, the lungs, spleens, kidneys and the blood of each infected mouse were homogenized and plated for colony counting. As reported in Fig. 7A, the number of wild-type bacteria was significantly higher than the number of mutant bacteria in all investigated organs. To confirm the results, we co-infected eight animals using equal numbers of wild-type and Δ*ap1*_M6 bacteria. After 24 h, lungs and spleens were dissected from each mouse and processed as described above. Wild-type and mutant bacteria were discriminated by colony blot assays using anti-AP1_M6 antibodies. As shown in Fig. 7B, calculation of the competitive index confirmed an approximate 10-fold difference in the number of wild-type versus Δ*ap1*_M6 colonies in both organs.

In order to investigate whether AP1_M6 contributes to bacterial survival in blood, wild-type bacteria, the deletion mutant Δ*ap1*_M6, and the complemented strain Δ*ap1*_M6 (pAM*ap1*_M6) grown to exponential phase, were incubated in the presence of whole blood from non-immunized rabbits. Bacterial survival was evaluated by comparing the initial bacterial input with the number of colony-forming units (cfu) after 5 h of incubation. Figure 7C shows the results of triplicate experiments expressed as the relative survival values of the two tested strains versus the wild type. As shown, the deletion mutant was unable to grow in blood, while growth was partially restored in the complemented mutant. Furthermore, addition of soluble rAP1_M6 protein to wild-type bacteria led to a decrease in growth, suggesting that AP1-mediated bacterial interactions contribute to blood survival.

## Discussion

It is well established that Gram-positive pili facilitate the initial contact of the bacteria with their host and the formation of biofilm-like three-dimensional structures. However, the detailed molecular interactions involved in this process have started to be deciphered only in recent times. The present study was aimed at investigating in detail the specific role of the AP1 ancillary pilus component in bacterial cell adhesion, biofilm formation and in the establishment of infection by GAS FCT-1 isolates.

In a first set of experiments, a contribution of the AP1_M6 protein to cell adhesion was suggested by the reduced capacity of in-frame deletion mutants to adhere to pulmonary epithelial cells compared with the parent strain. However, a definite conclusion could not be drawn from these results, as the *ap1*_M6 deletion also had a partial detrimental effect on the formation of BP_M6 pilus polymers. In further experiments, *L. lactis* expressing pili containing exclusively BP_M6 were shown to display the same adhesive capacity as the recipient strain, while adhesion was increased by about fourfold in *Lactococcus* expressing pili constituted by BP_M6 and AP1_M6; furthermore, a highly purified recombinant form of AP1_M6 protein displayed a strong cell binding affinity. Altogether the data led us to conclude that AP1_M6 is the main FCT-1 pilus adhesin.

*In silico* analysis revealed the presence of a Von-Willebrand factor type A domain (VWA), which mediates adhesion in several eukaryotic proteins via metal ion-dependent sites (Sadler, 1998), and that has been implicated in Group B *Streptococcus* PilA-mediated cell adhesion (Konto-Ghiorghi *et al*., 2009). Cell binding experiments using purified recombinant AP1_M6 fragments suggested that the VWA domain also confers adhesive properties to the GAS FCT-1 pilin. Notably, the VWA of PilA and AP1_M6 share less than 10% identity, suggesting that the two molecules bind to different host ligands. In a very recent publication it was demonstrated that binding of GBS PilA to the collagen extracellular matrix component promotes bacterial attachment to endothelial cells via α2β1 integrin, resulting in pro-inflammatory chemokine release (Banerjee *et al*., 2011). The identification of the molecular receptor of AP1_M6 in epithelial cells is the subject of our current investigations.

A peculiarity of FCT-1 pili is the absence of the small ancillary protein AP2, the least variable of the pilus components, which anchors the FCT-2 polymer to the cell wall peptidoglycan (Smith *et al*., 2010). Several types of evidence led us to exclude that AP1_M6 played a similar role in FCT-1 pili. First, the LPXTG motif typical of cell wall-anchoring sortase substrates was present in the M6 backbone protein but not in AP1, suggesting that the FCT-1 pilus was anchored through the backbone. Second, electron microscopy analysis localized the AP1 protein at the pilus tip or interspersed along the pilus, and only in some instances at its base. Finally, mild detergent treatment of Δ*ap1*_M6 did not result in the detachment and accumulation of pili in bacterial supernatants, as instead observed for deletion mutants of the pilus anchoring AP2 proteins of GBS (Nobbs *et al*., 2008) and GAS FCT-2 pili (Smith *et al*., 2010) (data not shown). In a recent study published during the preparation of this manuscript, Nakata and co-workers investigated the assembly mechanism of FCT-1 M6 pili by preparing knockout mutants of the pilus genes, and introducing modifications in amino acid residues possibly implicated in the covalent linkage of pilus polymers (Nakata *et al*., 2011). Consistent with our hypothesis, the authors demonstrated that the LPXTG motif of BP_M6 is essential for anchoring the FCT-1 pilus to the cell wall, while the LPSSG motif of AP1_M6 and the lysine residue of a novel pilin motif present in BP_M6 are necessary for incorporation of the ancillary protein to the pilus shaft.

The pioneering discovery of GAS pili in 2005 was concomitant with the demonstration of the protective capacity of vaccines constituted of pilus proteins in a mouse model of infection (Mora *et al*., 2005). Here we have shown that antibodies raised against rAP1_M6 are also capable of blocking biofilm formation. The data suggest that vaccines targeting the pilus proteins could also prevent colonization of two main GAS target tissues, the skin and the pharyngeal epithelia.

The specific role of AP1_M6 in the formation of large bacterial microcolonies became evident owing to the observation that the recombinant protein interfered with bacterial aggregation. These data also suggest the presence of a single binding site per AP1 molecule, as rAP1_M6 did not act as bridging molecule but instead prevented the formation of wild-type bacterial aggregates. Furthermore, the observation that recombinant rAP1_M6 bound to wild-type GAS FCT-1 M6 bacteria indicated that AP1_M6 homophilic interactions can take place when the proteins are embedded in a pilus structure. On the contrary, rAP1_M6 did not bind to the Δ*ap1*_M6 mutant strain, excluding interactions with other bacterial surface proteins. Overall, the data showed that homophilic interactions between AP-1 proteins from different individual microorganisms mediate inter-bacterial contact. This type of homophilic interactions were also observed for the AP1 moieties of FCT-2 and FCT-3 pili, leading us to propose a general mechanism by which these proteins contribute to the formation of bacterial aggregates on epithelial tissues serving as focal points of infection.

Homophilic interactions similar to those observed for the pilus AP1 ancillary proteins had previously been described for other microbial proteins involved in bacterial aggregation, biofilm formation and survival in blood, including *B. pertussis* haemagglutinin (Menozzi *et al*., 1994), staphylococcal Aap and Sas G (Rohde *et al*., 2005; Geoghegan *et al*., 2010), GAS protein H (Frick *et al*., 2000) and pneumococcal PsrP (Sanchez *et al*., 2010).

The possible role of AP1_M6 in GAS survival during infection was assessed in a mouse model of sepsis and in *ex vivo* growth experiments, where we observed that Δ*ap1*_M6 deletion mutants were outcompeted by wild type in target organs, and that their growth in blood was remarkably impaired.

Overall, the obtained results lead us to hypothesize that AP1 pilus components play a double role in GAS pathogenesis, by mediating both tissue colonization during early stages of disease through the formation of large cell-adhering microcolonies, and bacterial survival in more advanced stages of GAS infection.

## Experimental procedures

### Bacterial strains, recombinant proteins and antisera

*Streptococcus pyogenes* HRO-27_M6 was obtained from the University Hospital of Rostock (Germany). GAS bacteria were maintained on TSA agar plates supplemented with 5% defibrinated sheep blood and grown in liquid cultures at 37°C in Todd Hewitt Broth supplemented with 0.5% Yeast Extract or in C medium as described (Manetti *et al*., 2010). *L. lactis* MG1363 was grown at 30°C in M17 (Difco, Becton Dickinson, Rockville, MD, USA) supplemented with 0.5% glucose (GM17). *E. coli* DH5α and BL21(DE3) strains were grown in LB medium. Selective media contained 10 µg ml^−1^ Chloramphenicol or 1 µg ml^−1^ Erythromycin for GAS, 20 µg ml^−1^ Chloramphenicol for *L. lactis* and 20 µg ml^−1^ Chloramphenicol, 100 µg ml^−1^ Erythromycin or 100 µg ml^−1^ Ampicilin for *E. coli*.

The genes encoding AP1_M6, AP1_M6 fragments, BP_M6, AP-1_M1, AP1_M3 and Emm6 proteins were amplified from genomic DNA and cloned into the *E. coli* plasmid vector pET21 to produce 6XHis fusions as described (Manetti *et al*., 2010) except for AP1_M3 that was cloned into pGEXNNH (Montigiani *et al*., 2002) and was obtained as GST-His fusion. The recombinant fusion proteins were purified by affinity chromatography and specific antisera were obtained by immunizing CD1 mice and New Zealand rabbits with 20 µg of purified recombinant proteins formulated in Alum Hydroxide.

### Bacterial adhesion to epithelial cells

The human lung adenocarcinoma epithelial cell line A549 (ATCC CCL-185, Manassas, VA, USA) was cultured in Dulbecco's modified Eagle's medium supplemented with 10% fetal bovine serum (FBS) and 5 mM Glutamine (DMEM, Gibco BRL, Invitrogen Life Technologies, USA). Approximately 5 × 10^5^ cells were seeded into 12-well tissue culture plates and allowed to grow for 24 h. Bacteria grown to OD_600_ = 0.4 were used to infect the confluent cell monolayers using a bacterial: cell ratio of 100:1 for GAS and of 20:1 for *L. lactis*. After centrifugation for 30 s at 1000 r.p.m., the plates were incubated for 30 min at 37°C. Wells were extensively washed with PBS to remove unattached bacteria, treated with 1% saponin to lyse eukaryotic cells, and adherent bacteria were plated for enumeration. The average number of bacteria recovered per ml was determined for each single assay from three independent wells. Tests were repeated three times and the percentage of adhering bacteria versus total bacteria was calculated. The Student's *t*-test was used to determine the statistical significance of differences in cell adhesion between bacterial strains.

### Adhesion of recombinant proteins to epithelial cells

For Confocal Fluorescence Microscopy analysis of rAP1_M6 adhering to A549, approximately 5 × 10^5^ cells were seeded on glass coverslips placed in 12-well plates for 24 h in the presence of antibiotic-free DMEM with FBS 10%. Cells were incubated with 100 µg ml^−1^ rAP1_M6 protein for 2 h on ice. After that, wells were washed three times with 1× PBS and fixed with 2% PFA in PBS for 20 min at room temperature, washed twice with 1× PBS and incubated 1 h at 4°C with mouse anti-rAP1_M6 (1:3000 in PBS with 1% BSA). Cell-bound rAP1_M6 proteins were stained with Alexa Fluor 488-conjugated goat anti-mouse (Molecular Probes) diluted 1:5000 in PBS with 1% BSA. A549 cells were stained with Alexa Fluor 647-conjugated phalloidin (Molecular Probes) diluted 1:70 in PBS with 1% BSA (p/v) for 1 h at room temperature. Immunofluorescence images were recorded using a Bio-Rad Radiance 2000 Microscope and reconstructed from 0.5 mm confocal optical sections using VOLOCITY 3.6 (Improvisation, Lexington, MA, USA).

For FACS analysis of proteins binding to A549, cells were grown as described above, non-enzymatically detached from the support using cell dissociation solution (Sigma-Aldrich, USA), harvested and suspended in serum-free DMEM. Cells (1 × 10^6^) were mixed with increasing concentrations of each recombinant protein and incubated for 1 h at 4°C. After two washes with PBS, cells were incubated for 1 h at 4°C with mouse-specific polyclonal antisera diluted 1:200 in PBS. In control ELISA experiments, comparable specific antibody titres against each protein were obtained. After two washes, the samples were incubated on ice for 30 min with R-Phycoerythrin-conjugated goat antibody anti-mouse immunoglobulin (Jackson Immuno Research Laboratories), and cells were subsequently analysed using a FACS Calibur Flow-Cytometer and Cell Quest Software (Becton Dickinson). The net mean fluorescence intensity for each protein bound to cells was calculated as the obtained fluorescence signal minus the fluorescence intensity of the cells incubated with sera alone. A Scatchard analysis of the data obtained from three different experiments for rAP1_M6 was used to derive an apparent cell binding affinity (*K*_D_), as the concentration determining the saturation of 50% of the receptors present on the cells as previously described (Santi *et al*., 2007).

### Construction of GAS in-frame deletion and complemented mutants and of recombinant *L. lactis*

In-frame deletion mutants of HRO-27 M6 lacking the *ap1* gene were constructed by splicing-by-overlap-extension PCR as described (Mora *et al*., 2005). Briefly, in-frame deleted gene products were amplified using the following specific primers: M6AP-1F1: CTGAGGATCCAGGTGACAGATAATTCAGAGCAAGTA, M6AP-1R2: TGACTTCAATTCACCTTCAATCGGCTGCAAGCCTAGTCGCCTTAT, M6AP-1F3: ATAAGGCGACTAGGCTTGCAGCCGATTGAAGGTGAATTGAAGTCA, M6AP-1R4: CTGACTCGAGACCATTCCCTTTATCAGGCTTCTTA. The PCR product was cloned using BamHI and XhoI restriction sites in the temperature-sensitive shuttle vector pJRS233 (Perez-Casal *et al*., 1993). Transformation and allelic exchanges were performed as described (Perez-Casal *et al*., 1993) and drug-sensitive colonies were screened by PCR for the absence of the target allele.

To introduce the GAS FCT-1 pilus island into *Lactococcus*, the genomic regions between the start codon of the M6 gene *spy0159* (*ap1*_M6) and the stop codon of *spy0161*(*bp*_M6) or between the start codon of gene *spy0160* and *spy0161* were amplified using primers FpAM-pilusM6 CTGACTGAGCGGCCGCTTGAGAGGAGAGAAAATGTACAGTAGATTGAAGAGAGAGT plus RpAM-pilusM6 CTGACTGAGCGGCCGCCTCTCCTGTTTGTCAATAAATATGAT and FpAM-pliusM6Δ*ap1* CTGACTGAGCGGCCGCTTGAGAGGAGAGAAATTGACAAACAGGAGAGAAACAGTGA plus RpAM-pliusM6Δ*ap1* CTGACTGAGCGGCCGCCTCTCCTGTTTGTCAATAAATATGAT respectively. To complement the Δ*ap1*_M6 mutant, the *ap1* gene was amplified using primers FpAM-*ap1*_M6 GCTCGGATCCTCTTGCTTGGATGACTACTTAGAAC plus RpAM-*ap1*_M6 GCTCGGATCCTAATATT GCTTTTTCCTGTGTTGGCGT. All PCR products were cloned into the shuttle vector pAM401 as described (Buccato *et al*., 2006) using NotI and BglII restriction sites, and the obtained constructs pAM-pilusM6, pAM-pilusM6Δ*ap1* and pAM*ap1*_M6 were introduced in *L. lactis* MG1363 competent cells by electroporation.

### Immunoblot and Far Western blot assays

GAS cell wall-enriched fractions were prepared as previously described (Manetti *et al*., 2010), loaded onto 3–8% gradient SDS-PAGE (Invitrogen Life Technologies, USA) and transferred to nitrocellulose membranes (Whatman, GE, USA). The amount of the different protein extracts loaded on gels were normalized using antibodies to Spy0269, as described (Manetti *et al*., 2010). The membranes were incubated with mouse-specific polyclonal antisera at a 1:1000 dilution, horseradish peroxidase-linked anti-mouse IgG secondary antibody (Dako Cytomation, Denmark) at a 1:5000 dilution, and developed with a chemiluminescence detection substrate (Super-Signal West Pico, Pierce, Rockford, USA).

For Far Western blot assays, recombinant proteins were size-separated by SDS-PAGE (4–12% gel) and blotted onto nitrocellulose membranes. Immunostaining was performed by blocking the membrane overnight with 3% (w/v) skimmed milk in TPBS and incubating for 1 h with biotinylated recombinant proteins (EZ-Link Sulfo-NHS-LC-LC-Biotin, Pierce, Rockford, USA). After washing, the membrane was subsequently incubated for 1 h with HRP-conjugated Streptadivin (Invitrogen Life Technologies, USA) and then developed with a chromogenic substrate.

### Immunogold labelling and electron microscopy

Immunoelectron microscopy experiments were performed as described (Rosini *et al*., 2006). GAS and *L. lactis* strains from overnight cultures were grown to exponential phase. Bacteria were then centrifuged for 10 min at 3000 *g*, washed and suspended in PBS. Formvarcarbon-coated nickel grids were floated on drops of GAS suspensions for 5 min. Bacteria adhered to the grids were fixed in 2% Paraformaldehide for 15 min at 22°C and incubated with Blocking Solution (PBS containing 1% normal mouse serum and 1% BSA) for 30 min at 22°C. The grids were then floated on drops of primary antiserum against GAS proteins diluted 1:20 in blocking solution for 30 min, washed with blocking solution, and floated on secondary antibody conjugated to 5 or 10 nm gold particles diluted 1:10 in 1% BSA for 30 min. After washing with PBS and with four drops of distilled water and air dried, the grids were examined using a TEM JEOL 1200EX II transmission electron microscope at 80 kV and a CCD camera (Megaview, Eloise, USA).

### Biofilm and aggregation assays

Biofilm formation was investigated by crystal violet stained plate assays as described (Manetti *et al*., 2007). Briefly, bacterial overnight cultures were diluted in fresh C medium and incubated for 10 h at 37°C in 24-well plates. Subsequently the medium was removed, wells were extensively washed with PBS, and adherent bacteria were stained with 0.2% crystal violet. After washing with PBS, crystal violet was recovered with 1% SDS and the biomass was quantified at 540 nm. Analyses were performed in triplicate in three distinct experiments and the mean and standard deviation results are presented. To perform biofilm inhibition assays, equal titres of specific polyclonal sera were added to the wells prior to bacterial incubation. All experiments were run in triplicate, and statistical differences between samples were calculated by the Student's *t*-test.

For bacterial sedimentation assays, GAS wild type and its Δ*ap1*_M6 mutant were grown overnight in 5 ml of C-medium in optical tubes, in the absence or in the presence of 1 µM recombinant rBP_M6 or rAP1_M6. Test tubes were gently inverted to suspend the bacterial cells and the initial optical density value at 600 nm was measured. The bacterial cultures were then left to settle at 22°C and sedimentation was evaluated by collecting the OD values every hour for 6 h without shaking, as an indication of bacterial aggregation.

### FACS analysis of wild-type and mutant bacteria

The levels of surface expression of AP1_M6, BP_M6, Emm6 and ScpA proteins in wild-type and Δ*ap1*_M6 GAS strains was evaluated by FACS analysis using mouse-specific antibodies. Bacteria were grown to OD_600_ = 0.4, washed twice with PBS, suspended in newborn calf serum (Sigma) and incubated for 20 min at room temperature. Cells were subsequently incubated on ice for 30 min with pre-immune or immune mouse sera at a final dilution of 1:200 in PBS/0.1% BSA. Bacteria were washed, incubated on ice for 30 min in 10 µl of goat anti-mouse IgG conjugated with F(ab′)2 fragment-specific R-Phycoerythrin (Jackson Immunoresearch Laboratories) in PBS/0.1% BSA/20% newborn calf serum to a final dilution of 1:100. Bacteria were then fixed by incubating for 15 min at 22°C with 2% formaldehyde (Sigma-Aldrich, USA), washed in PBS, and analysed by FACSCalibur flow cytometer (BD Bioscences, Rockville, MD, USA) and a CellQuest Software (Becton Dickinson). The fluorescence of the bacteria stained with pre-immune or immune sera was illustrated by two-dimensional overlaid histograms representing fluorescence intensities calculated with voltage adjustment at FL2 linear channel.

### FACS analysis of rAP1_M6 binding to bacteria

Purified rAP1_M6 was labelled with biotin using the EZ-Link Sulfo-NHS-LC-LC-Biotin kit (Pierce, Thermo Scientific, Rockford, IL, USA) according to manufacturer instructions. GAS cells were grown to exponential phase (OD_600_ = 0.4), washed with PBS, suspended in FBS (Invitrogen Life Technologies, USA) and incubated for 20 min at 22°C. Bacteria were then washed with buffer 1 (0.1% BSA in PBS) and incubated for 1 h at 4°C with (or without) 5 µg of biotynilated rAP1_M6 in buffer 1. After washing with buffer 1, samples were stained for 1 h at 4°C with R-Phycoerythrin-conjugated Streptavidin (Southern Biotech, USA) and washed with buffer 1 and PBS. Bacteria were then fixed by incubating for 15 min at 22°C with 2% formaldehyde (Sigma-Aldrich, USA), washed in PBS and analysed by a FACSCalibur flow cytometer (BD Bioscences, Rockville, MD, USA). For *L. lactis* experiments, bacteria were fixed in 0.5% formaldehyde prior to incubation with biotinylated rAP1_M6. Data were evaluated using CellQuest Software by acquiring 10 000 cellular events per sample file. Comparison between the fluorescence of the bacteria stained with biotinylated rAP1_M6 with that of unstained bacteria were illustrated by two-dimensional overlaid histograms representing fluorescence intensities calculated with voltage adjustment at FL2 linear channel. FACS results were also expressed as delta mean fluorescence intensity (ΔMFI) of each histogram, where ΔMFI was calculated by subtracting the mean fluorescence of the bacteria stained with biotinylated recombinant rAP1_M6 protein from that of unstained bacteria.

### ELISA analysis of homophilic rAP1_M6 interactions

Homophilic rAP1_M6 interactions were analysed by ELISA as previously described (D'Angelo *et al*., 2005; Margarit *et al*., 2009). Microtitre plates were coated overnight at 4°C with 1 µg of rAP1_M6 per well in 50 mM sodium carbonate pH 9.5, and treated for 1 h at 22°C with PBS 2% BSA. The plates were then incubated for 1 h with increasing amounts of biotinylated rAP1_M6. After extensive washing, plates were incubated for 20 min with HRP-conjugated Streptadivin (Invitrogen Life Technologies, USA). After washing, bound conjugated enzyme was reacted with a chromogenic substrate, and absorbance at 490 nm was determined. To calculate the relative affinity association constant (*K*_A_) value of the interaction between biotinylated rAP1_M6 and the immobilized unlabelled protein, the data were fitted using the following equation: *A* = Amax [*L*] *K*_A_/(1 + *K*_A_ [*L*]), where [*L*] is the molar concentration of biotinylated ligand. The reported dissociation constant (*K*_D_ value) was calculated as reciprocal of the *K*_A_. The assay was performed three times with reproducible results.

### *In vivo* GAS infection

Wild-type and Δ*ap1*_M6 GAS strains were grown to OD_600_ = 0.4, washed in PBS and resuspended in THY medium. In three independent experiments, four CD1 mice were infected intraperitoneally with 2 × 10^7^ WT bacteria and four CD1 mice with 2 × 10^7^Δ*ap1*_M6. After 24 h, lungs, spleens, kidneys and blood were homogenized and plated in duplicate at various dilutions. Statistical differences between the number of WT versus Δ*ap1*_M6 cells were calculated using the Mann–Whytney *U*-test.

For co-infection experiments, two groups of four CD1 mice received a bacterial suspension containing an equal number of 10^7^ WT and 10^7^Δ*ap1*_M6 cells. After 24 h, lungs and spleens were processed as above. Colony blots using antisera against rBP_M6 were performed to distinguish between the survival frequencies of the two strains. All animal studies were performed according to guidelines of the Italian Istituto Superiore di Sanità and Novartis Animal Welfare.

### Whole blood survival assays

Wild-type strain, its deletion mutant Δ*ap1*_M6 and the complemented strain Δ*ap1*_M6(pAM*ap1*_M6) were grown to exponential phase (OD_600_ = 0.4). In the case of the complemented strain, Chloramphenicol was added to growth medium to help maintaining the plasmid. Bacteria were harvested by centrifugation, suspended in DMEM (Gibco, Invitrogen Life Technologies, USA), serially diluted in the same medium and plated for bacterial enumeration. The bacterial suspension (50–100 cfu) was incubated 15 min on ice with DMEM, with or without 2 µM rAP1_M6 protein. Subsequently, the bacterial reaction was incubated end-over-end (20 r.p.m.) with whole blood from non-immunized rabbits at 37°C, 5% CO_2_. After 5 h, the reactions were serially diluted and plated for enumeration. Bacterial growth rate in blood was calculated as the ratio between the number of cfu after 5 h incubation (cfu at time 5 h) and the initial input (cfu at time 0). For each strain, the average relative growth rate (cfu at time 5 h versus time 0) versus that of wild type (to which we assigned a value of 100) obtained from three different experiments and its standard deviation were reported. The Student's *t*-test was used to determine the statistical significance of the observed differences.
