# Supplementary material for: The ancillary protein 1 of Streptococcus pyogenes FCT-1 pili mediates cell adhesion and biofilm formation through heterophilic as well as homophilic interactions
Source: Mol Microbiol. 2012 Feb 10;83(5):1035–47. doi: 10.1111/j.1365-2958.2012.07987.x (PMC3490378; doi:10.1111/j.1365-2958.2012.07987.x)

SUPPLEMENTARY MATERIAL FOR ONLINE PRESENTATION

**Supplementary Figures 1 and 2**

**Fig. S1. Flow cytometry analysis of WT_M6 and Δ*ap1*_M6 strains.** Wild-type (panel A) and Δ*ap1*_M6 bacteria (panel B) were incubated with mouse polyclonal antibodies raised against rAP1_M6, rBP_M6, rEmm6 and rScpA proteins and stained with R-Phycoerythrin conjugated goat anti-mouse secondary antibodies. Black histograms indicate staining of bacteria with pre-immune serum, while colored histograms correspond to bacteria stained with specific sera.

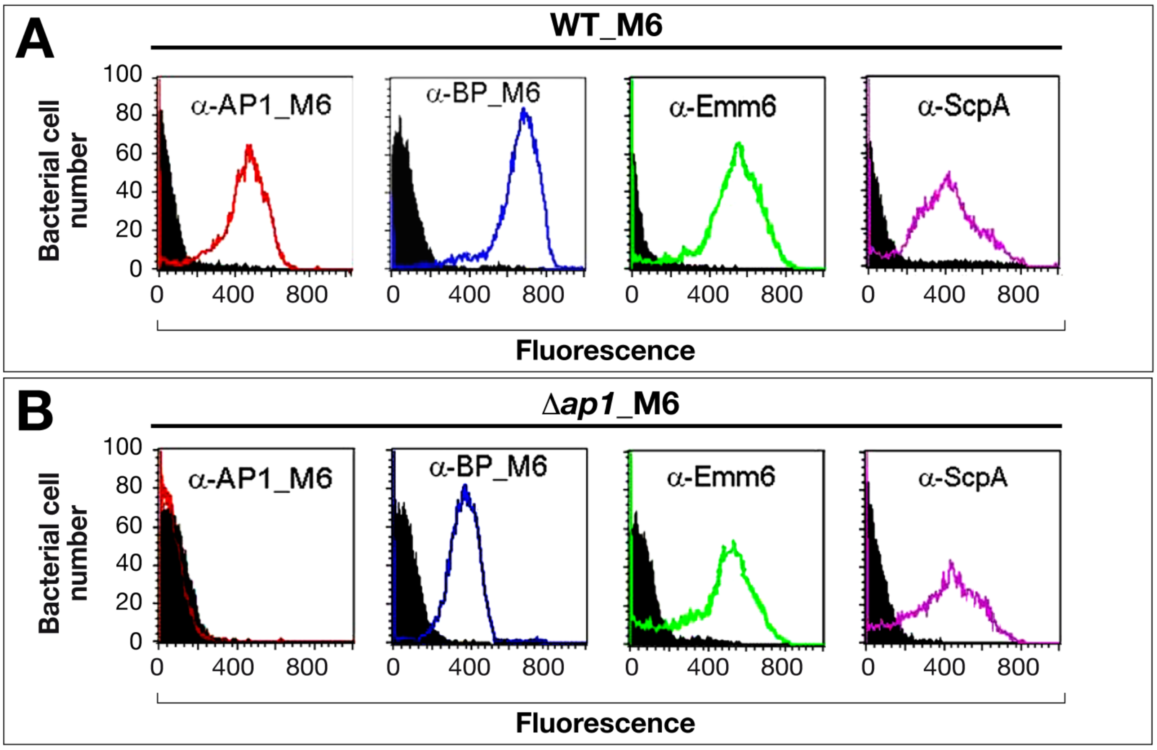

**Fig. S2. Binding of rAP1_M6 fragments to epithelial cells.**

A) SDS PAGE analysis of purified recombinant pilus protein rAP1_M6 and its three fragments: an N-terminal portion spanning residues 1 to 499, a C-terminal fragment from aminoacids 505 to 1068 including the VWA and the Cna_B domains, and a shorter C-terminal fragment from aminoacids 964 to 1068 containing exclusively the Cna_B domain. B) FACS analysis of rAP1_M6 and its three fragments binding to A549 cells. Cells were incubated with increasing concentrations of the recombinant proteins and cell-bound proteins were detected with antibodies raised against each of the polypeptides and fluorescent secondary antisera. Results are presented as the mean and standard deviation values of 3 independent experiments.

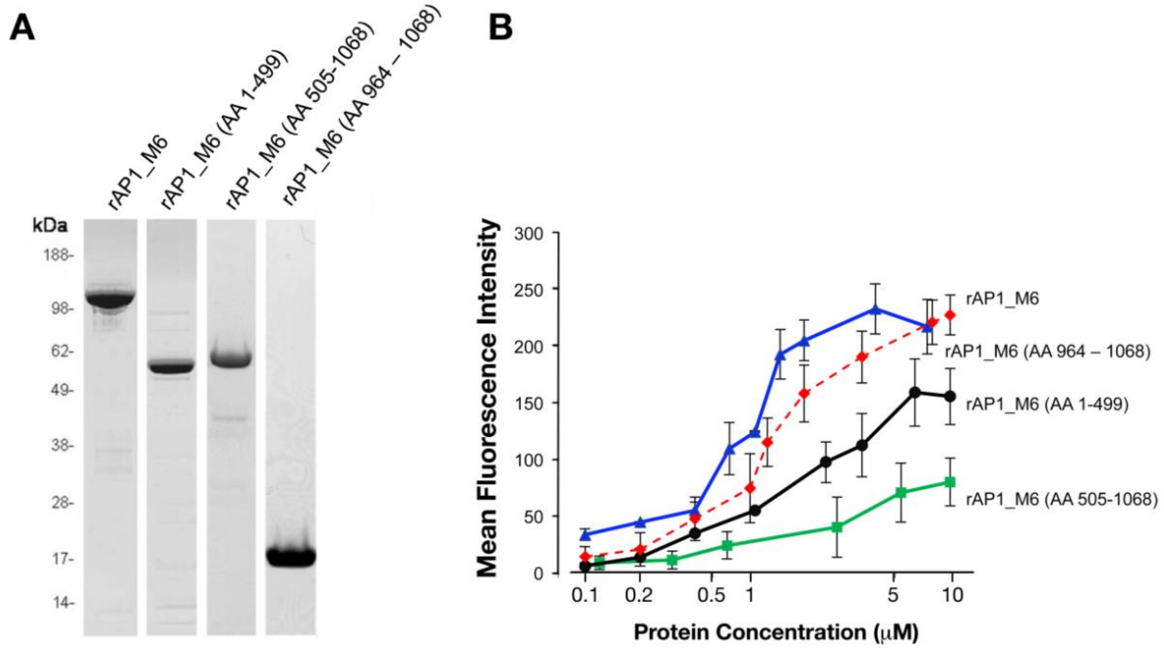

Supplement: Supplementary file 1 [file mmi0083-1035-SD1.pdf]
